# Longitudinal Three-Dimensional Follow-Up and Secondary Treatment Aspects after Endoscopic and Open Scaphocephaly Surgery

**DOI:** 10.1097/PRS.0000000000010701

**Published:** 2023-05-16

**Authors:** Guido A. de Jong, Jene W. Meulstee, Erik J. van Lindert, Wilfred A. Borstlap, Thomas J. J. Maal, Hans H. K. Delye

**Affiliations:** Nijmegen, the Netherlands; From the 1 Department of Neurosurgery; 2 Radboudumc 3D Lab; 3 Department of Oral and Maxillofacial Surgery, Radboud University Medical Center.

## Abstract

**Background:**

This retrospective cohort study evaluated the longitudinal three-dimensional cranial shape developments and the secondary treatment aspects after endoscopically assisted craniosynostosis surgery (EACS) with helmet therapy and open cranial vault reconstruction (OCVR) for scaphocephaly.

**Methods:**

Longitudinally collected three-dimensional photographs from scaphocephaly patients and healthy infants were evaluated. Three-dimensional cranial shape measurements and growth maps were compared between the groups over time. Secondary treatment aspects were compared for the treatment groups.

**Results:**

Both surgical techniques showed their strongest changes directly after surgery, with mean parietal three-dimensional growths up to 10 mm. At age 24 months, comparison of head shapes showed mean three-dimensional differences less than ±2 mm, with OCVR resulting in a lower vertex and longer cranial length when compared with EACS. At 48 months of age, no measurements were significantly different between treatment groups. Only the total head volume was somewhat larger in the male EACS group at age 48 months (*P* = 0.046). Blood loss in EACS (mean, 18 mL; range, 0 to 160 mL) was lower than in OCVR (mean, 100 mL; range, 15 to 300 mL; *P* < 0.001). Median length of stay after surgery was shorter for EACS (mean, 2 days; range, 1 to 5 days) compared with OCVR (mean, 5 days; range, 3 to 8 days; *P* < 0.001).

**Conclusions:**

The authors conclude that EACS for scaphocephaly shows equal craniometric results at age 48 months and has a better surgery profile compared with OCVR. Early diagnostics and referral for suspected scaphocephaly to allow EACS is therefore recommended.

**CLINICAL QUESTION/LEVEL OF EVIDENCE:**

Therapeutic, III.

Scaphocephaly is the result of premature fusion of the sagittal suture and is characterized by an elongated head, a wide and prominent forehead, and a narrow occiput.^1^ Scaphocephaly is the most common type of craniosynostosis and occurs in 1.5 to 4 of 10,000 live births worldwide.^2–5^

Treatment of scaphocephaly aims to correct cranial deformities, prevent increased intracranial pressure, and reduce the risk of developmental delay.^6^ Among the wide variety of treatment options, open cranial vault reconstruction (OCVR) is the most common method, while minimal invasive treatment options such as spring mediated cranioplasty and endoscopically assisted craniosynostosis surgery (EACS) in combination with helmet therapy increase in popularity.^6–9^ It is expected that these techniques could result in different cranial shape developments after surgery.

Studies show that the endoscopic technique reduced complication and mortality rates, decreased blood loss, shortened hospital stay, and lowered health care costs.^3,7,10–12^ Aesthetic treatment outcomes are often described with simple two-dimensional measures such as the cephalic index (CI) and subjective measures which fail to give an adequate description of the cranial shape.^13,14^ Three-dimensional stereophotogrammetry in combination with advanced evaluation methods can overcome these limitations and allow objective long-term follow-up of the patients’ cranial morphology.

The aim of this study was to evaluate the cranial shape development of scaphocephaly patients with objective three-dimensional analysis methods. In addition, the secondary treatment aspects were evaluated.

## PATIENTS AND METHODS

Ethical approval from the regional institutional review board was obtained (no. 2020-6128). All consecutive unisutural nonsyndromic scaphocephaly patients aged up to 56 months who underwent either EACS or OCVR between 2005 and 2019 at our institute were included. Patients older than 14 months at the time of surgery are rare in our institution and were excluded.

### Treatment

For children younger than 6 months, EACS is performed in our center.^8^ During EACS, the fused sagittal suture is removed trough 2 small skin incisions with the aid of the endoscope, and biparietal stave osteotomies are performed. Helmet therapy starts 2 weeks after EACS for all patients and continues for a period of approximately 10 months. During this period, every 3 months a three-dimensional photograph is made to evaluate head growth and adjust/change/stop the helmet therapy if needed.

For children older than 6 months at referral, OCVR is performed in our institution. Since 2013, a virtual surgical plan is created that provides the opportunity to virtually manipulate the skull segments and visualizes the effect of the osteotomies. Various operation strategies can be simulated, which avoids unnecessary or inadequate osteotomies during the actual surgery and helps to establish the best surgical outcome for each patient. To transfer the surgical planning to the patient, specific drawing templates are used to demarcate the osteotomy lines and assembly templates are used for a swift reconstruction of the skull cap. Resorbable plates (SonicWeld, KLS Martin, Tuttlingen, Germany) are used for fixation of the cranial segments. No helmet therapy was indicated for OCVR patients. (**See Figure, Supplemental Digital Content 1**, which shows notable steps in EACS from our institution. [*Above, left*] Skin incision lines with schematic overview of affected suture cutting lines. [*Above, right*] External view of minimally invasive removal of affected suture and additional osteotomies. [*Below, left*] Endoscopic view of minimally invasive removal of affected suture and additional osteotomies. [*Below, right*] Resected fused sagittal suture and biparietal side cuts, http://links.lww.com/PRS/G807. **See Figure, Supplemental Digital Content 2**, which shows notable steps in OCVR from our institution. Before surgery, the reconstruction is virtually planned and executed by our team to determine the optimal positions of the skull segments and osteotomies. Patient-specific surgical guides are used to transfer the plan to the patient. [*Left*] Drawing osteotomy lines on the exposed skull using the guides. [*Center*] Positioning of skull segments in a specific mold before fixation. [*Right*] Repositioning of expanded skull segment, fixed in place using resorbable plates [SonicWeld], http://links.lww.com/PRS/G808.)

### Three-Dimensional Photographs

Three-dimensional photographs of patients were acquired using the 3DMD Cranial System (3dMDCranial; 3dMD, Atlanta, GA). The three-dimensional photographs of patients who underwent treatment were distributed in the nearest age group (3, 6, 9, 12, 15, 18, 24, 36, and 48 months) and distributed in the before-surgery and after-surgery groups, depending on the timing of surgery.

### Three-Dimensional Photograph Processing

The same data acquisition, processing, and alignment protocols were followed for the three-dimensional photographs as described in earlier work.^15–17^ All three-dimensional photographs underwent quality control and were excluded in case of insufficient quality and were aligned using the Computed Cranial Focal Point (CCFP) method to allow objective evaluation.^16,18^

The scaphocephaly CCFP-offset values were determined using 20 before-surgery computed tomography (CT) scans of scaphocephaly patients.^16^ Reference values were similarly acquired using CT scans of infants aged 0 to 48 months.^15^ Presurgical three-dimensional photographs were positioned using the scaphocephaly-specific CCFP-offset. Both the postsurgical OCVR three-dimensional photographs and the reference group three-dimensional photographs were positioned using the reference age-specific CCFP-offset values as the head shape of the after-surgery OCVR group was being modeled toward normal shaped heads. The after-surgery EACS three-dimensional photographs used scaphocephaly CCFP-offset values interpolated toward the reference value from 6 to 18 months of age. After 18 months, the reference CCFP-offset values were used for the EACS three-dimensional photographs.

### Three-Dimensional Photograph Measurements

For all three-dimensional photographs, the cranial length was measured from the most anterior point to the most posterior point of the cranium and the cranial width was determined by a line perpendicular to the cephalic length.^15^ Dividing cranial width by cranial length resulted in the CI. Circumference was measured on the cranial shapes at the crossing points of the cranial width and length. The volume above the sella turcica to nasion plane was calculated. Results were split on sex, type of surgery, and before or after surgery status.

### Growth Maps

Color-coded growth and shape comparison maps were created to visualize the shape changes of the head and used to compare the groups. To counter any non–craniosynostosis-related asymmetries of the head, the three-dimensional photographs were mirrored and averaged over the midsagittal axis before analysis. These growth maps visualize the difference between the mean head shapes in two sequential age groups or between two treatment groups. Patients were grouped based on sex, age, and treatment. Finally, to investigate the long-term outcome, the results of both surgical strategies were compared at 24 months of age.

### Secondary Treatment Aspects

Secondary treatment aspects included the following: age at surgery, sex, blood loss, surgery time, total anesthesia time, blood transfusions (during and after surgery), hospital stay duration, and intensive care unit (ICU) stay duration. Helmet therapy duration was also obtained for EACS.

### Statistical Analysis

For all three-dimensional photograph measurements, the mean and standard error of mean were computed per data set per group and a linear mixed model analysis was performed. The intervention group, age group, and the interaction between intervention group and age group, were chosen as fixed effects. Random effects were specified to the individual cases and intercept. The mixed model analysis was performed for each three-dimensional measurement and sex.

### Two-Tailed *t* Tests for Three-Dimensional Measurements between Age Groups per Intervention and Gender Were Conducted

Means and standard deviations were given for normally distributed data. Medians and the range were given for nonnormally distributed data. If an outcome value was normally distributed in one subgroup but was not in the counterpart group, both values were reported with medians and ranges for consistency. Level of statistical significance was set at *P* < 0.05. For statistical analyses, IBM SPSS Statistics version 25 (IBM Germany GmbH, Ehningen, Germany) was used.

## RESULTS

### Three-Dimensional Photograph Acquisition

Altogether, 384 three-dimensional photographs from 106 EACS patients, and 108 three-dimensional photographs from 34 OCVR patients were used. The EACS and OCVR groups contained 84 and 29 male patients, respectively. A total of 130 reference three-dimensional photographs with 64 male patients (49%) up to the age of 24 months were obtained from an earlier study.^15^ The three-dimensional photograph distribution is shown in Table 1.

**Table 1.** Number of Three-Dimensional Photographs per Age Group before and after Surgery for EACS and OCVR Groups^a^

Group	Age (mo)	Before Surgery				After Surgery				References	
		EACS		OCVR		EACS		OCVR			
		M	F	M	F	M	F	M	F	M	F
1	3	52	13	4	1	4	2			14	12
2	6	18	3	8		33	3	1	1	10	12
3	9			8		47	11	4		13	14
4	12			8	2	47	13	7		10	9
5	15					26	6	9	1	4	7
6	18					12	3	10	3	10	8
7	24					37	8	17	2	3	4
8	36					8	2	4	2		
9	48					29	7	15	1		

M, male; F, female.

a All groups were divided into male and female patients.

### Secondary Treatment Aspects

The secondary treatment aspects of 114 EACS patients (92 male patients) and 36 OCVR patients (29 male patients) were collected and shown in (Table 2). The male-to-female ratio was 4:1 in both treatment groups. EACS patients had less blood loss, shorter surgery, shorter anesthesia time, and a shorter length of stay. Only one EACS patient was treated in the ICU for 3 days. In the OCVR group, 25 patients were admitted to the ICU for 1 day each. The remodeling helmet was worn 9.6 ± 2.4 months on average (range, 3.9 to 16.1 months) after EACS.

**Table 2.** Overview of the Surgical Safety Parameters between the EACS and OCVR Groups and Their Respective Differences as Indicated by the *P* Values

Parameter	Group		
	EACS (%)	OCVR (%)	*P*
Sex			0.985
Male	92 (81)	29 (81)	
Female	22 (19)	7 (19)	
Age, mo			<0.001
Median	3.9	9.2	
Range	2.4–6.6	4–14	
Blood loss, mL			<0.001
Median	18	100	
Range	0–160	15–300	
Blood transfusion perioperative and postoperative	21 (19)	29 (81)	<0.001
Total amount transfused, mL			<0.001
Median	90	130	
Range	65–190	30–250	
Length of stay after surgery, days			<0.001
Median	2	5	
Range	1–5	3–8	
Total length of stay total, days			<0.001
Median	3	5	
Range	2–7	4–9	

Fewer blood transfusions were given during surgery in EACS, and the amount of blood given was significantly lower in the EACS group. Remarkably, in both OCVR and EACS groups, the postsurgical transfusion rate was the same (*n* = 7 [19%] and *n* = 19 [17%], respectively).

### Three-Dimensional Measurements

**Fig. 1.**
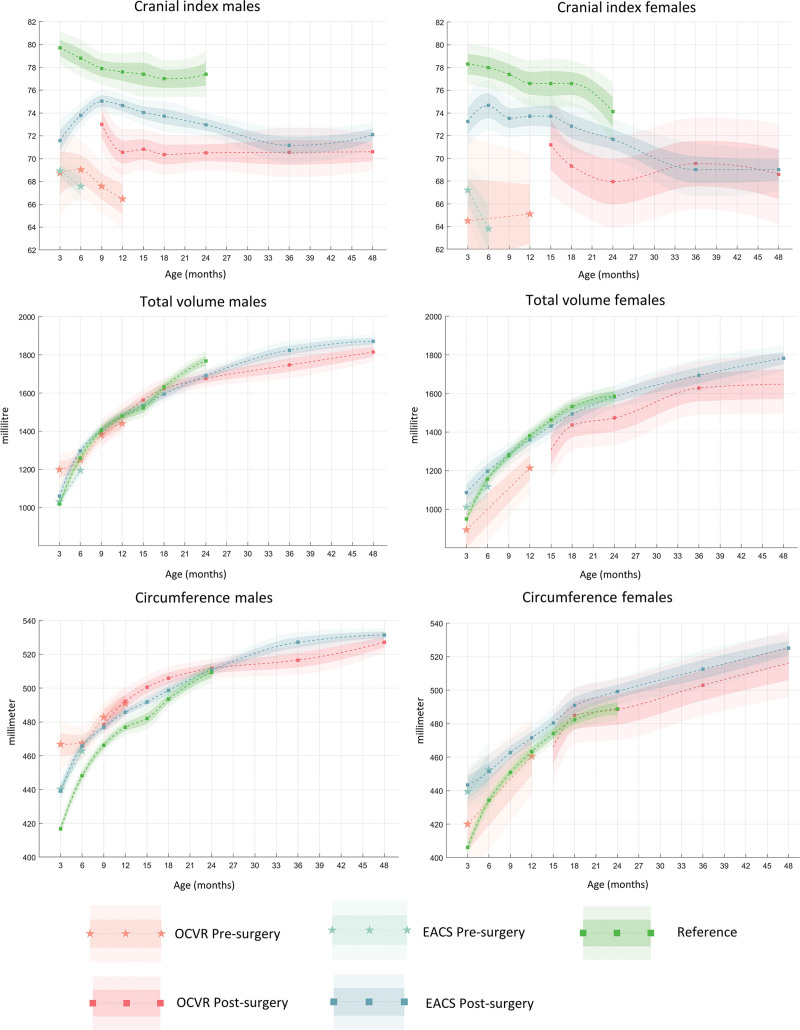
Graphs of the mean and standard error of mean of the cephalic index, total cranial volume and circumference after EACS and OCVR for scaphocephaly at given ages in months.

The before-surgery and after-surgery measurements are shown in Tables 3 through 6. The graphs and standard deviations of CI, total volume, and circumference is shown in Figure 1. The majority of the before-surgery values were significantly different from the reference groups in both genders.

**Table 3.** Before-Surgery Shape Measurements of the Male Scaphocephaly Patients

Group	No.	Age (mo)	Cranial Width (mm)		Cranial Length (mm)		CI		Circumference (mm)		Total Volume (mL)	
			Mean	SEM	Mean	SEM	Mean	SEM	Mean	SEM	Mean	SEM
EACS 1	52	3	111^a^,^b^	1	161^a^	1	68.9^b^	0.5	440^a^,^b^	2^a^	1030^a^	13
EACS 2	18	6	115^b^	1	170^b^	1	67.6^b^	0.8	463^b^	3	1195^b^	20
OCVR 1	4	3	117^a^	3	171^a^,^b^	3	68.7^b^	1.9	467^a^,^b^	7	1199^a^,^b^	45
OCVR 2	8	6	118^b^	2	171^b^	2	69.0^b^	1.3	467^b^	5	1249	31
OCVR 3	8	9	120^b^	2	178^b^	2	67.6^b^	1.1	483^b^	4	1381	28
OCVR 4	8	12	121^b^	2	182^b^	2	66.5^b^	1.3	491^b^	5	1441	32

a Significant difference between surgery groups.

b Significant difference with healthy references.

**Table 4.** After-Surgery Shape Measurements of the Male Scaphocephaly Patients

Group	No.	Age (mo)	Cranial Width (mm)		Cranial Length (mm)		CI			Circumference (mm)		Total Volume (mL)	
			Mean	SEM	Mean	SEM	Mean	SEM	Δ Pre	Mean	SEM	Mean	SEM
EACS 1	4	3	114	1	160^b^	2	71.6^b^	0.9	+3.0	439^b^	4	1060	29
EACS 2	33	6	123	1	167^a^,^b^	1	73.8^b^	0.5	+5.2	466^b^	2	1297	16
EACS 3	47	9	128	1	171^b^	1	75.0^b^	0.5	+6.5	477^b^	2	1408	15
EACS 4	47	12	130	1	174^a^,^b^	1	74.6^a^,^b^	0.5	+6.1	486^b^	2	1482	15
EACS 5	26	15	131	1	177^a^,^b^	1	74.0^a^,^b^	0.5	+5.5	492^a^	2	1534	17
EACS 6	12	18	132^b^	1	180^a^	1	73.7^a^,^b^	0.7	+5.1	499	3	1594	20
EACS 7	37	24	134^b^	1	185	1	73.0^a^,^b^	0.5	+4.4	511	2	1692^b^	16
EACS 8	8	36	136	1	192	1	71.2	0.8	+2.6	527^a^	3	1825	24
EACS 9	29	48	139	1	193	1	72.1	0.5	+3.5	531	2	1872^a^	16
OCVR 2	1	6	125	3	176^a^,^b^	4	70.8^b^	2.1		483	9	1376^b^	67
OCVR 3	4	9	126	2	173^b^	2	73.0^b^	1.2	+5.2	478^b^	5	1388	37
OCVR 4	7	12	127^b^	1	179^a^,^b^	2	70.6^a^,^b^	1.0	+2.7	492^b^	4	1480	31
OCVR 5	9	15	130	1	183^a^,^b^	2	70.8^a^,^b^	0.9	+3.0	501^a^,^b^	3	1563	27
OCVR 6	10	18	131^b^	1	185^a^,^b^	2	70.4^a^,^b^	0.9	+2.5	506^b^	3	1624	27
OCVR 7	17	24	132^b^	1	187^b^	1	70.5^a^,^b^	0.7	+2.7	512	3	1677^b^	23
OCVR 8	4	36	134	2	189	2	70.6	1.1	+2.7	517^a^	4	1747	34
OCVR 9	15	48	137	1	193	1	70.6	0.8	+2.8	527	3	1815^a^	25

Δ Pre, changes from the before-surgery group.

a Significant difference between surgery groups.

b Significant difference with healthy references.

**Table 5.** Before-Surgery Shape Measurements of the Female Scaphocephaly Patients

Group	No.	Age (mo)	Cranial Width (mm)		Cranial Length (mm)		CI		Circumference (mm)		Total Volume (mL)	
			Mean	SEM	Mean	SEM	Mean	SEM	Mean	SEM	Mean	SEM
EACS 1	13	3	109	2	162^b^	2	67.2^b^	1.1	439^b^	5	1011^b^	27
EACS 2	3	6	107^b^	3	168^b^	4	63.8^b^	2.1	452^b^	9	1117	53
OCVR 1	1	3	101	6	156	6	64.5	3.7	420	16	893	92
OCVR 4	2	12	112^b^	4	172	5	65.1^b^	2.6	461	11	1214^b^	65

a Significant difference between surgery groups.

b Significant difference with healthy references.

**Table 6.** After-Surgery Shape Measurements of the Female Scaphocephaly Patients

Group	No.	Age (mo)	Cranial Width (mm)		Cranial Length (mm)		CI			Circumference (mm)		Total Volume (mL)	
			Mean	SEM	Mean	SEM	Mean	SEM	Δ Pre	Mean	SEM	Mean	SEM
EACS 1	2	3	117^b^	2	161^b^	3	73.3^b^	1.2	+6.7	443^b^	6	1086^b^	42
EACS 2	3	6	121	2	163^b^	3	74.7	1.1	+8.1	451^b^	5	1196	40
EACS 3	11	9	123	1	168^b^	2	73.5^b^	0.9	+7.0	463^b^	4	1283	29
EACS 4	13	12	125	1	170^b^	2	73.7^b^	0.8	+7.1	472^b^	4	1361	29
EACS 5	6	15	128^a^	1	174^b^	2	73.7	0.9	+7.2	480	4	1431	33
EACS 6	3	18	129	2	178^b^	2	72.8^b^	1.1	+6.3	491	5	1494	38
EACS 7	8	24	131^a^	1	183^b^	2	71.7	0.9	+5.1	499^b^	4	1583	31
EACS 8	2	36	130	2	189	3	69.0	1.2	+2.4	513	6	1694	42
EACS 9	7	48	133	1	193	2	69.0	0.9	+2.4	525	4	1783	32
OCVR 5	1	15	121^a^,^b^	3	170	5	71.2	2.2		466	10	1308^b^	76
OCVR 6	3	18	124^b^	3	179^b^	4	69.3^b^	1.9	+4.4	485	8	1437	64
OCVR 7	2	24	123^a^,^b^	3	181	4	67.9^b^	2.0	+3.0	488	9	1473	69
OCVR 8	2	36	129	3	185	4	69.5	2.0	+4.6	503	9	1628	69
OCVR 9	1	48	131	3	191	5	68.6	2.2		516	10	1648	77

Δ Pre, changes from the before-surgery group.

a Significant difference between surgery groups.

b Significant difference with healthy references.

The average presurgical CI for male patients was 68.6 for EACS and 67.8 for OCVR. For the female population, these values were 66.6 for EACS and 64.9 for OCVR. Both treatment groups showed an increase of the CI from before to after surgery; however, CI was higher in the reference groups. The EACS patients showed an increase in CI in the first months after surgery for both sexes, followed by a decline at approximately 9 to 12 months. OCVR patients have a constant postsurgery CI over time (approximately 69 to 71). The CIs of EACS patients showed a decline to approximately that of the OCVR CI values over time, which finally resulted in no significant differences between these groups.

Cranial width varied but had no clear pattern regarding the significant differences between the surgery groups themselves, and between the surgery groups and references. However, cranial width was significantly smaller for EACS and OCVR patients before surgery compared with the references, but this difference resolved after surgery for both groups. The cranial length in both groups remained longer compared with the references.

The circumference of both treatment groups was significantly larger than the references in the earlier after-surgery age groups. The volume differences between the treatment groups and the references were minimal.

### Growth Maps

**Fig. 2.**
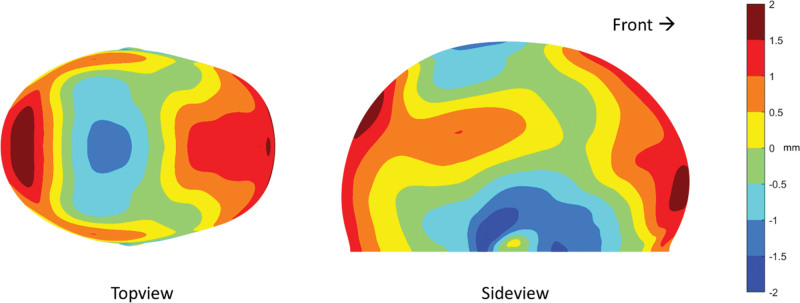
Growth maps indicating the mean head shape differences (in millimeters) between EACS and OCVR at 24 months of age (after surgery). The EACS head shape is baseline; thus, a positive value indicates that the OCVR head shape has localized additional volume over that of EACS and vice versa. Note the color scaling (0.5 mm per unique color). The length of the EACS head shape is shorter and the vertex is higher and the bitemporal distance larger.

The most important growth map is the comparison at 24 months between treated EACS and OCVR patients (Fig. 2). The maximum differences are within the −2 mm and +2 mm range. Typically, OCVR treated patients have a more elongated head with a growth focus around the frontal and occipital areas, a somewhat narrow lower temporal region, and a slightly wider lower parietal region. Furthermore, the vertex of OCVR patients is slightly lower. Two examples of children aged 24 months, treated using EACS or OCVR, are shown. (**See Figure, Supplemental Digital Content 3**, which shows [*above*] a three-dimensional photograph of a 24-month-old child treated with EACS. [*Below*] A three-dimensional photograph of a 24-month-old child treated with OCVR. From *left* to *right*: top view, side view [*right*], front view, http://links.lww.com/PRS/G809.)

**Fig. 3.**
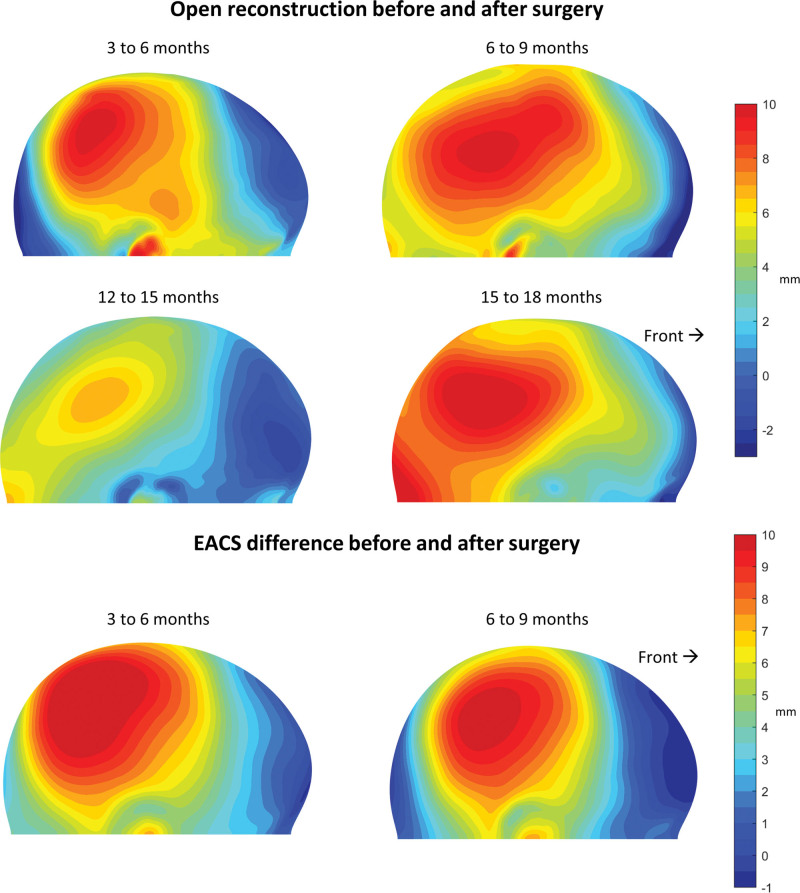
(*Above*) Growth maps indicating the mean head shape differences (in millimeters) from before to after EACS surgery for those aged 3 to 6 months and 6 to 9 months. (*Below*) Growth maps indicating the mean head shape differences (in millimeters) from before to after OCVR surgery for those aged 3 to 6 months until 15 to 18 months. Note the color scaling (0.5 mm per unique color).

The most important findings of the growth maps are reported below and visualized in Figures 2 through . The growth maps of EACS patients from before to after surgery for age groups 1 (3 months) and 2 (6 months) both show a strong parietal growth of up to 10 mm (Fig. 3). The frontal and occipital growth for the first age group were approximately 0 and 4 mm, respectively. For the second age group, frontal and occipital growth was less compared with the earlier surgery.

**Fig. 4.**
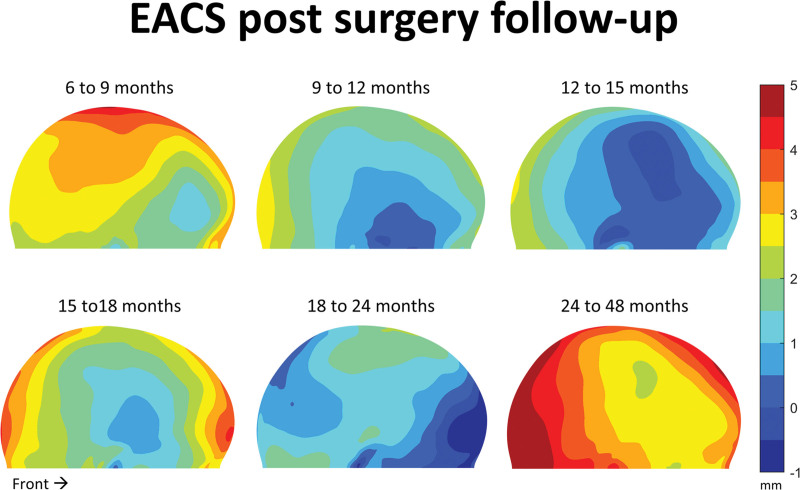
Growth maps indicating the mean head shape differences (in millimeters) after EACS surgery for 3 to 6 months until 24 to 48 months of age. Note the color scaling (0.5 mm per unique color).

In OCVR, parietal changes up to 10 mm can be seen regardless of the age at surgery (Fig. 3). Nevertheless, OCVR also showed differences between before and after surgery, depending on the age during surgery. In contrast to EACS, occipital growth up to 7 mm is present between 6 and 9 months; frontal changes remain limited in all age (OCVR) groups. Occipital growth is more present in OCVR for later surgery ages as compared with the earlier surgery ages.

The after-surgery follow-ups for EACS patients are shown in Figure 4. From 6 to 9 months of age, a prominent upper parietal growth of 5 mm is noted, with lower parietal growth of approximately 3 mm. Frontal areas grow up to 4 mm. The frontotemporal region only shows 1 to 2 mm of growth. From 9 to 12 months, only 0 to 1 mm of frontotemporal growth, up to 3 mm of occipital growth, and a midline growth up to approximately 2.5 mm were noted. Nearly identical growth is present between 9 and 18 months. However, growth at 18 months is more prominent around the occipital and frontal area (up to 5 mm). Between 18 and 24 months, only frontoparietal growth and some lower occipital growth up to 2.5 mm is present. Between 24 and 48 months, some frontotemporal growth (up to 3 mm) is dominant.

## DISCUSSION

### Secondary Treatment Aspects

In general, EACS performed equal to or better than the OCVR in all aspects of the secondary treatment. Only one EACS patient was admitted to the ICU because of a non–craniosynostosis-related issue. This suggests that there were no EACS-induced ICU admittances, in contrast to 25 cases for OCVR.

Curiously, the OCVR blood loss levels in this study seem lower than reported in other studies.^19–22^ We think that the use of virtual surgical planning techniques in our institute, reducing the overall surgical time and blood loss, could be a contributing factor. Our findings are in line with, and add up to, the growing bulk of reports showing the superior safety profile of minimally invasive techniques such as EACS over open remodeling techniques.^3,11,19–31^

### 3D Measurements

Cranial width shows a strong postsurgical increase (especially in EACS). This results in a CI incline in the first months after EACS for both sexes, followed by a decline at approximately 9 to 12 months. OCVR patients have a constant after-surgery CI over time (approximately 69 to 71). The trend of CI over time is in line with other longitudinal studies.^21,28,32^

The decline of CI around 9 to 12 months in the EACS group co-occurs (in most cases) with the stopping of helmet molding therapy. Because a similar decline in CI in the reference group at age 9 to 12 months can be noted, this suggests a natural growth pattern rather than a relapse after stopping helmet therapy.

Overall, CI in the EACS group remains higher than in the OCVR group, and is significantly different for earlier age groups. For later age groups, the effect diminishes in both sexes, with a CI approximating 70.

Before-surgery studies report CI values in the range of 67 to 70, which is in line with our findings.^21,31^ Most of the after-surgery CI values for scaphocephaly correction remain in the 75 to 85 range, which are higher than ours.^7,12,21,28,31,33–35^ This may be attributable to a demographic difference in the patient population or to the measuring method.^10,36^

The longitudinal circumference changes did not differ between OCVR and EACS and were in line with literature.^7,28^ When compared with the normal reference group, we found that in the early age groups, circumference data are larger in scaphocephaly patients, but at 24 months, the circumference has obtained normal proportions again, for both treatment groups.

Volume measurements differ per study because of the lack of consensus on volume measuring methods.^37^ Nevertheless, taking measuring differences into account, we found similar volume changes for OCVR as reported by others.^35,38^ The volume data of EACS patients are very similar to those of our reference group^15^ and the OCVR group. Therefore, our data show that both techniques result in equal and normal intracranial volumes.

### Growth Maps

The growth maps of both EACS and OCVR groups (Figs. 3 and 4) show an impressive increase in cranial growth. A clear increase of lateral expansion and vertex height can be noticed, resulting in a proper lateral profile for both treatment options. In Figure 2, the cranial length of the OCVR is greater compared with the EACS group. This can be explained by the fact that OCVR patients are treated at a later age and thus have longer and larger compensatory growth in the anteroposterior direction. The vertex height of the EACS group is higher than that of the OCVR group and showed a natural and appealing spherical shape of the cranium. This contrasts with Le et al., who reported that EACS results in a lower vertex height compared with OCVR.^7^ This is an interesting finding, and although we did not measure head height, this could be a valuable additional three-dimensional measurement^7,10,13,39,40^ when comparing different surgical strategies (conventional or minimally invasive), such as spring-mediated cranioplasties.^35,41,42^

During the first months after EACS, the major effect of the growth appears to expand and enlarge the head (Figs. 2 and 3). After these first 3 to 6 months, this expanding effect, mainly present in the cranial width, reaches its limits. However, the helmet therapy is continued on average until approximately 10 months after surgery. Based on the results, it is uncertain whether the helmet therapy still benefits the outcome after this 3- to 6-month period. As stated before, when looking at the growth maps of 9 to 12 months and 12 to 15 months, growth seems to be oriented anteroposteriorly, resulting in a decline of CI at approximately 9 to 12 months. This could be because of the stopping of helmet therapy at that time, but a similar anteroposterior growth orientation is seen in growth maps of normal references at the same age.^15^ This suggests that perhaps this growth pattern resembles a natural predefined pattern and, instead of having a relapse, patients may rather shift toward a normal cranial growth pattern after EACS and helmet therapy. It would be interesting to see whether other groups could confirm these findings, as this might suggest that early reopening of a fused suture could invoke restoration of the normal growth potential of the head. In contrast, helmet therapy seems to increase CI, regardless of being implemented before or without surgery, although the latter raises concerns regarding intracranial pressure.^34,43–45^

### Limitations

With this being a retrospective, single-center study, there were methodologic limitations, such as selection bias. We tried to limit this by including all consecutive patients in the given time frame, randomizing between EACS or OCVR based solely on the age at first presentation in our center. This resulted in two surgical groups that were not significantly different in terms of demographic data and preoperative shape measurements (Tables 3 and 5). Because of our institution’s preference to perform EACS if possible, data for OCVR cases were limited. It was therefore difficult to create meaningful long-term longitudinal growth maps for OCVR.

Although this study used an extensive data set, it was not always possible to collect three-dimensional photographs on the exact time points because of logistic, technological, or patient-specific reasons, and this resulted in noncontinuous follow-up. Furthermore, the limited amount of three-dimensional photographs and unequal gender distribution was the reason why sexes were combined in the three-dimensional growth maps. These limitations could be overcome in a multicenter, prospective, randomized trial.

Despite three-dimensional photography being abundantly used and verified for certain applications in three-dimensional cranial analysis, we must remain cautious when comparing different measuring modalities such as CT scans and three-dimensional photographs or caliper measurements. Each measuring methodology has its own strengths and weaknesses, and results cannot be easily compared between modalities. Because there are no large normative data sets on cranial shape measurements obtained by three-dimensional stereophotogrammetry available in the literature, we could not calculate and compare *Z* scores. However, because we only compare data in EACS, OCVR, and a reference group that is acquired and analyzed similarly, we feel that our data are valid.

The designs of the remolding helmets and the patient-tailored surgical plans have a very important effect on the final cranial shape. Nevertheless, because it is almost impossible to take these factors into consideration and differentiate further, we decided to group the patients and evaluate the combined outcomes of the patients in the two treatment groups.

Finally, remodeling techniques vary greatly between centers. Therefore, the results of this study are center-specific and we would encourage other centers to perform similar analyses using their surgical OCVR technique. Nevertheless, the long-term evaluation and secondary treatment aspects used in this study confirmed our preference for EACS with helmet therapy.

## CONCLUSIONS

Given our data and the treatments options used in our center, we conclude that EACS before the age of 6 months is a valid treatment option for the correction of scaphocephaly, as it attains the same surgical results as OCVR up to the age of 48 months in our center. We consider this the preferred treatment option for scaphocephaly, because of better secondary treatment aspects. Early diagnostics and referral for suspected craniosynostosis to make EACS a viable option is therefore recommended.

## DISCLOSURE

The authors declare they have no financial relationships or conflict of interest to disclose.

## ACKNOWLEDGMENT

Several of the authors of this article are members of the European Reference Network CRANIO (project identification no. 739543).

## Supplementary Material


