# Supplementary figures and images for: Longitudinal Three-Dimensional Follow-Up and Secondary Treatment Aspects after Endoscopic and Open Scaphocephaly Surgery
Source: Plast Reconstr Surg. 2023 May 16;154(1):189–201. doi: 10.1097/PRS.0000000000010701 (PMC11195932; doi:10.1097/PRS.0000000000010701)

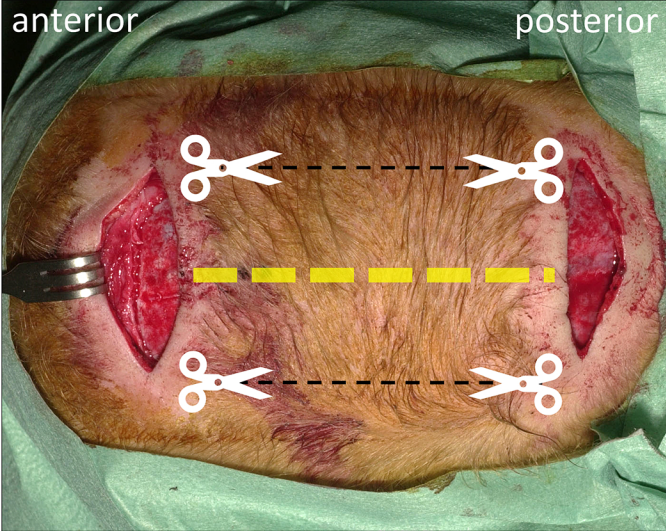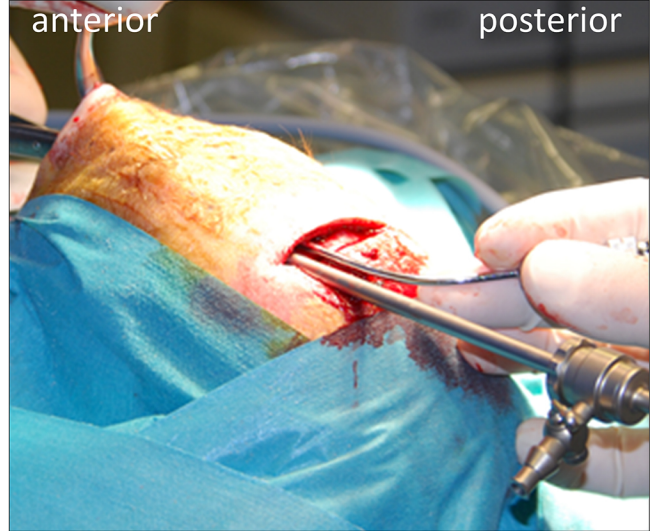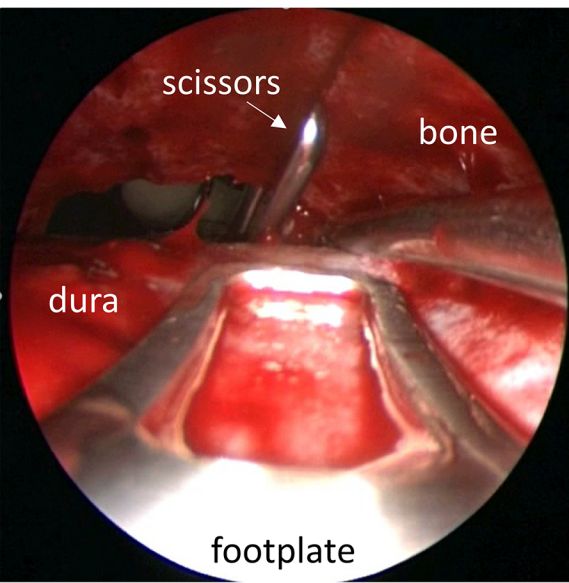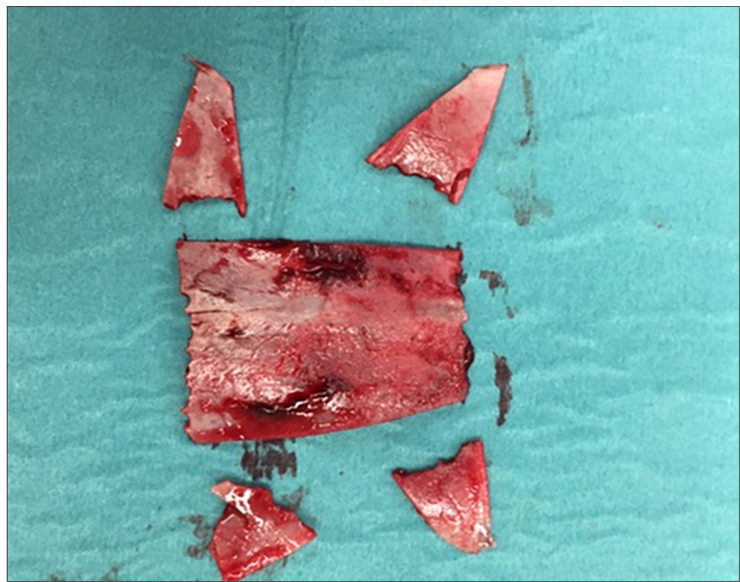

Supplement: Supplementary file 1 [file prs-154-0189-s001.pdf]

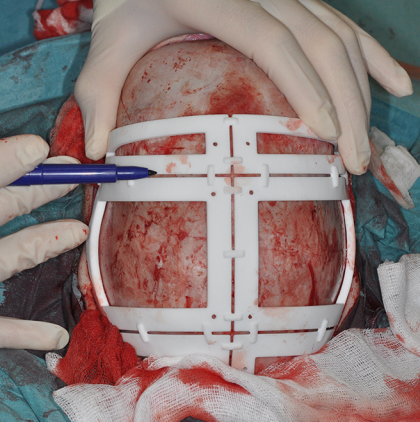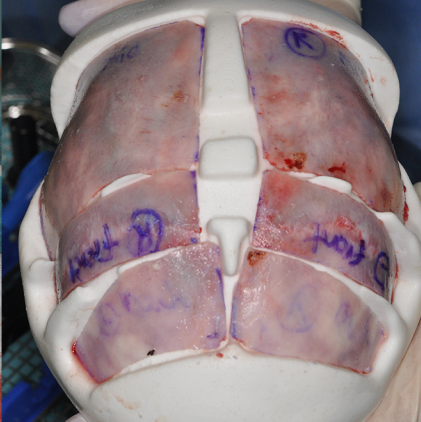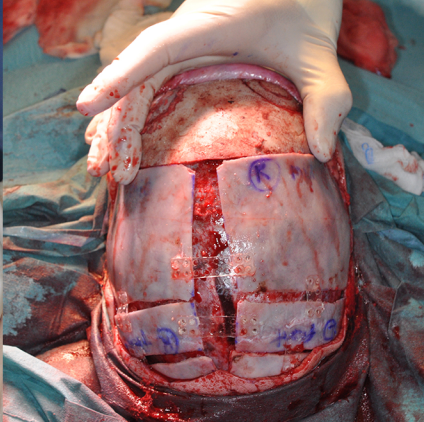

Supplement: Supplementary file 2 [file prs-154-0189-s002.pdf]

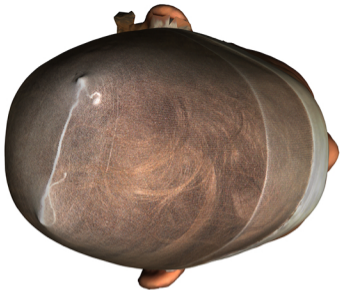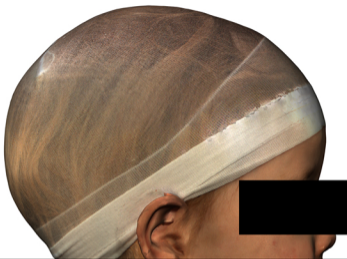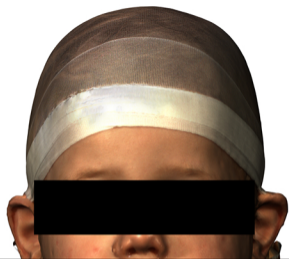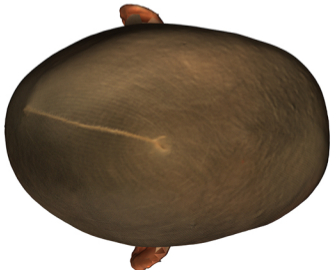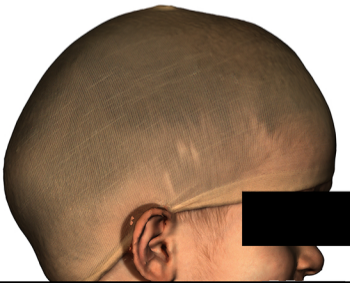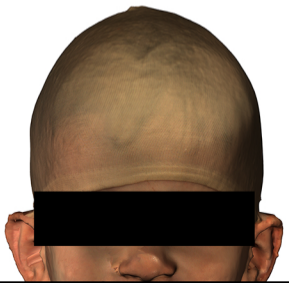

Supplement: Supplementary file 3 [file prs-154-0189-s003.pdf]
